# Topically applicated curcumin/gelatin-blended nanofibrous mat inhibits pancreatic adenocarcinoma by increasing ROS production and endoplasmic reticulum stress mediated apoptosis

**DOI:** 10.1186/s12951-020-00687-2

**Published:** 2020-09-05

**Authors:** Tao Cheng, Zhiheng Zhang, Hua Shen, Ziying Jian, Junsheng Li, Yujun Chen, Yi Shen, Xinyi Dai

**Affiliations:** 1 Department of General Surgery, Zhongda Hospital, Medical School, Southeast University, Nanjing, 210000 China; 2 Department of Surgery, Klinikum Rechts Der Isar, School of Medicine, Technical University of Munich, 81675 Munich, Germany; 3 Department of Plastic Surgery, Shanghai General Hospital, Shanghai Jiao Tong University School of Medicine, Shanghai, 200080 China; 4 Department of Hematology and Oncology, Zhongda Hospital, Medical School, Southeast University, Nanjing, 21000 China; 5 Bio-ID Center, School of Biomedical Engineering, Shanghai Jiao Tong University, Shanghai, 200240 China; 6 Department of Plastic and Reconstructive Surgery, Shanghai Ninth People’s Hospital, Shanghai Jiao Tong University, School of Medicine, Shanghai, 200011 China

**Keywords:** Curcumin, Nanofibrous mat (NM), Pancreatic adenocarcinoma, Tumor suppression, Bioavailability

## Abstract

**Background:**

Pancreatic adenocarcinoma (PDAC) is one of the most fatal malignancies. Surgical resection supplemented by chemotherapy remains the major therapeutic regimen, but with unavoidable resistance and systemic toxic reaction. Curcumin is a known safe natural compound that can effectively eliminate pancreatic adenocarcinoma cells in vitro, making it a promising candidate for substitution in subsequent chemotherapy. However, due to its extremely low bioavailability caused by its insolubility and circular elimination, curcumin had an unexpectedly modest therapeutic effect in clinical trials.

**Results:**

Here, we electrospun curcumin/gelatin-blended nanofibrous mat to largely improve curcumin’s bioavailability by local controlled-release. With characterization by scanning electron microscopy, fluorescence microscopy, Fourier transform infrared spectroscopy, X-ray diffraction and high-performance liquid chromatography, it was revealed that curcumin was uniformly dispersed in the fiber of the mats with nanoscopic dimensions and could be continuously released into the surrounding medium for days. The cancer inhibitory effects of nano-curcumin and underlying mechanisms were further explored by assays using pancreatic adenocarcinoma cell and experiments using xenograft model. The results showed the released nano-curcumin could effectively inhibit pancreatic adenocarcinoma cell proliferation not only in vitro, but more importantly in vivo. This cytotoxic effect of nano-curcumin against pancreatic adenocarcinoma was achieved through provoking the production of intracellular reactive oxygen species and activating endoplasmic reticulum stress, which leads to enhanced cell apoptosis via decreased phosphorylation of signal transducer and activator of transcription 3.

**Conclusions:**

Clinically, curcumin/gelatin-blended nanofibrous mat could be a promising, secure, efficient and affordable substitutional agent for the elimination of residual cancer cells after tumor resection. Moreover, our strategy to obtain curcumin released from nanofibrous mat may provide a universally applicable approach for the study of the therapeutic effects and molecular mechanisms of other potential medicines with low bioavailability.
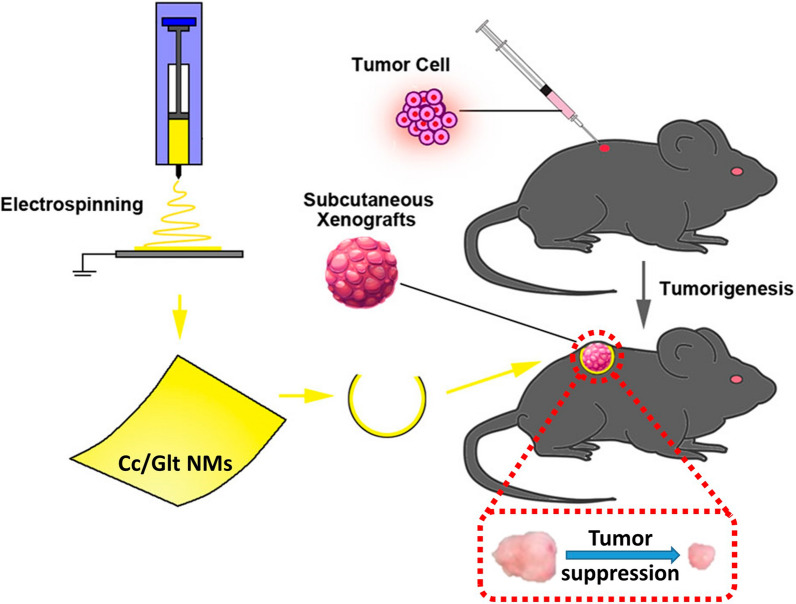

## Background

Pancreatic cancer is one of the most lethal malignancies, with an estimated 458,918 new cases diagnosed worldwide and an associated 432,242 deaths in 2018, which presents a considerable health problem [1–4]. Among them, pancreatic adenocarcinoma (PDAC) is the most malignant subtype with a 5-year survival rate of less than only 5% [5]. Radical operation is the only way to cure PDAC, but over 85% of patients are already at advanced stages upon initial diagnosis that has lost the chance of radical resection [6–8]. Currently, coalition therapy combining surgery with chemotherapy, radiotherapy or immunotherapy is the main option in the clinic, however, PDAC is characterized by an extraordinary resistance to chemo- and radiotherapy as well as to molecularly targeted therapy and immunotherapy [9–17]. Worse still, the nonspecific damage caused by radiotherapy as well as chemotherapeutic agents’ toxicity-related side effects further reduce patients’ life quality [18–30]. Besides, for most patients, especially those in developing countries, immunotherapy or chemotherapy is simply not available or not affordable [31–38]. Therefore, physicians and scientists are in constant search for alternative therapeutic agents that is more safe, efficient and cost-effective.

Recently, curcumin (chemical formula: C_21_H_20_O_6_; (1E,6E)-1,7-Bis(4-hydroxy-3- methoxyphenyl)-1,6-heptadiene-3,5-dione), the main bio-therapeutic compound in turmeric, has been identified and shown its great potential to induce apoptosis of various types of cancer cells [39–43]. In addition to its excellent pharmaceutical effects, curcumin has a broad range of sources and has been verified to have extremely low toxicity to human, as a reported safe dose of curcumin in clinical trials can be as high as 8000 mg/day, rendering it a promising candidate for combined chemo-therapy against pancreatic cancer immediately after ablative surgery [44–47]. However, despite the fact that curcumin is safe and inexpensive with significant pharmacological actions in vitro, its performance against pancreatic cancer in the clinic is unexpectedly modest [46, 48]. This is mainly due to its inherent defects of insolubility, instability as well as its poor absorption and rapid systemic elimination via systematic administration [44, 47, 49, 50]. What’s more, because of its low bioavailability and potency, it is difficult to gain reliable evidence from laboratory studies to fully elucidate the molecular mechanisms of curcumin against pancreatic cancer. Although, several types of curcumin analogs can be obtained by structural modification. Some of these analogs have been shown with improved solubility and have been used to investigate potential mechanisms of curcumin’s cytocidal impact on pancreatic cancer cell. However, the molecular structure of these analogs is obviously different from that of natural curcumin. Therefore, it is uncertain whether the discovered mechanisms of curcumin analog apply to curcumin itself. Moreover, the biosafety of these analogs has not been verified by long-term practice, so they are still a long way to the clinical translation [51, 52]. Altogether, to address the problems of low bioavailability and potency of curcumin itself may be a more direct and efficient mean of curcumin being further translated as favorable pharmacotherapy against pancreatic adenocarcinoma. In the case of removing residual cancer cells after ablative surgery, a suitable carrier system capable of sustained local drug delivery, ideally as surgical implant, seems to be an adoptable strategy, as it has been shown to be effective in dealing surgical wounds [53, 54].

In this work, we proposed a method to significantly enhance the bioavailability of curcumin by electospinning of curcumin/gelatin-blended nanofibrous mats (NMs). Therefore, curcumin is dispersed by encapsulated inside the NMs to improve its solubility by optimized release kinetics. By topical delivery, low doses of curcumin released from curcumin/gelatin NMs (Cc/Glt NMs) has been shown to efficiently eliminate PDAC cells without additional cytotoxic effect on normal somatic tissue cells. Meanwhile, as a fiber-forming material, gelatin possesses good biodegradability, non-antigenicity and pliability, so the prepared curcumin/gelatin NM has favorable biocompatibility and can be easily applied topically to surgical margins left out from tumor resection, which avoids fast curcumin elimination via systematic administration and minimizes non-targeted drug delivery to cause additional damage to normal tissue [55]. The in vitro and in vivo experiments showed that curcumin/gelatin NMs could induce PDAC programmed cell death and suppress tumor growth. In addition, we demonstrated that released curcumin activated the downstream endoplasmic reticulum stress (ER stress) signaling pathway (Bip/p-PERK/p-elF2a) and impaired phosphorylation of signal transducer and activator of transcription 3 (STAT3). Given these, together with the safety, potency and economy of curcumin and the good biodegradability and non-antigenicity of gelatin, the fabrication of curcumin/gelatin nanofibrous mat could be a promising alternative for targeted chemo-therapy after ablative surgery against not only pancreatic adenocarcinoma but also many other neoplastic diseases. Moreover, the strategy developed here, which disperse the low solubility molecules into nanofibers to make them can be sustained released with a high concentration level, may also be adopted as a universal approach for the investigation of potential molecular mechanisms of other therapeutic biomolecules with low bioavailability.

## Results

### Curcumin dispersion and controlled release from NMs

**Fig. 1:**
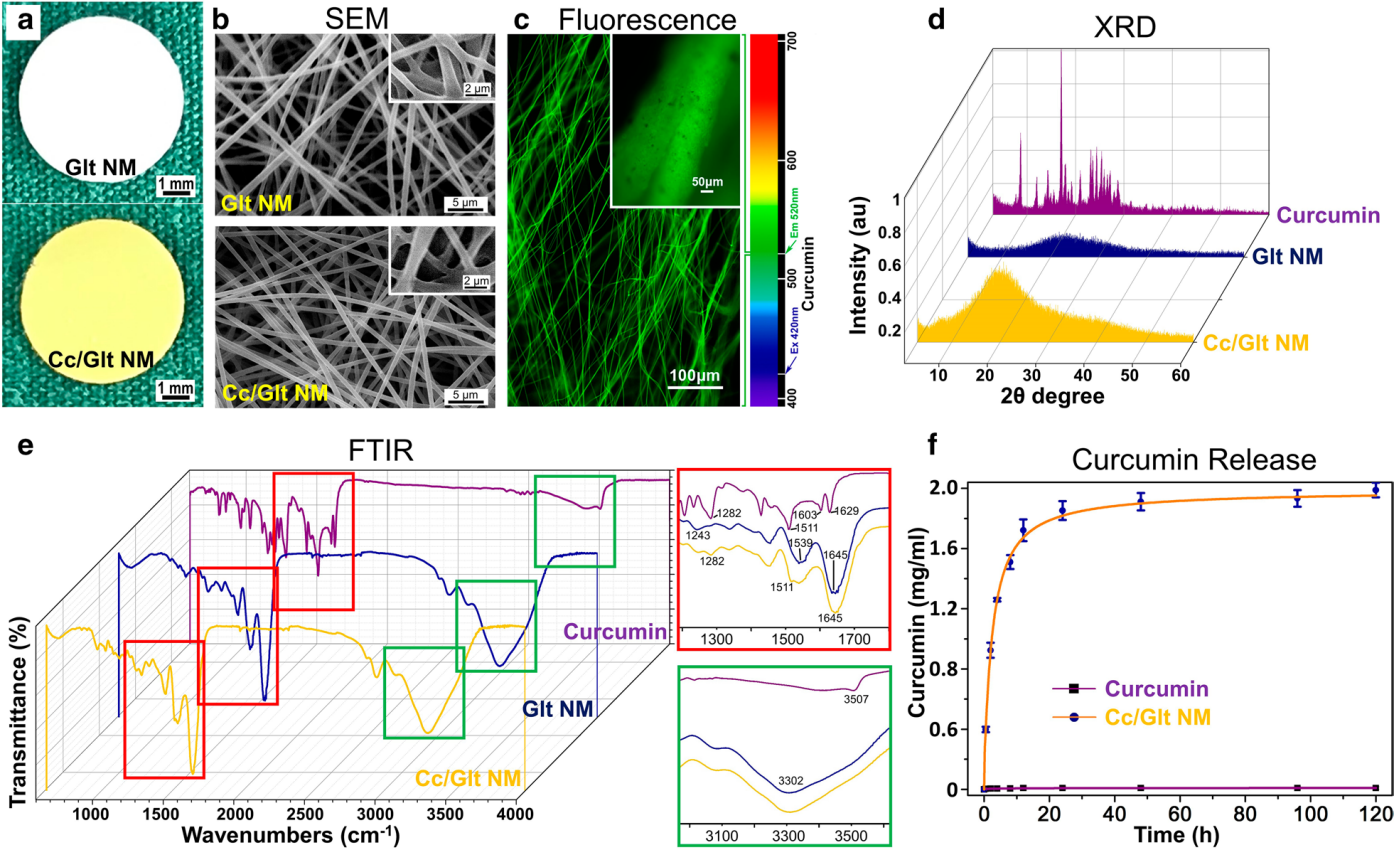
In vitro characterization of Cc/Glt NMs and controlled release of curcumin. **a** Photographs of the prepared Glt NM (upper) and Cc/Glt NM (lower); **b** scanning electron microscopy images of the detailed fiber structures of Cc/Glt NM; **c** fluorescence microscopy image of the curcumin distribution in the nanofiber as well as in the nanofibrous mat; **d** XRD spectra of curcumin in Cc/Glt NMs; **e** FTIR spectra of curcumin in Cc/Glt NMs; and **f** in vitro release curve of curcumin from Cc/Glt NMs (error bar: standard deviation)

A gelatin and curcumin mixture was electrospun into a millimeter-scale nanofibrous mat and cross-linked with glutaraldehyde. The color of the electrospun NMs changed markedly from white to yellow after the addition of curcumin (Fig. 1a). By scanning electron microscopy (SEM) and inverted fluorescence microscopy, the detailed fiber structures of the NMs with and without curcumin were imaged. The appearance of smooth fiber surface as well as the uniform fluorescence intensity of the Cc/Glt NMs indicates a homogeneous distribution of curcumin in the nanofibrous mat without aggregates larger than 100 nm (Fig. 1b, c). The structure of curcumin in the Cc/Glt NMs were further examined by X-ray diffraction (XRD) spectroscopy. The typical characteristics of crystalline curcumin shown as sharp intense peaks at various diffraction angles, were not seen in the Cc/Glt NMs, which means that curcumin in the Cc/Glt NMs is formulated as amorphous solid dispersion (Fig. 1d). We also employed Fourier transform infrared spectrometry to explore the state of curcumin in Cc/Glt NMs (Fig. 1e). The results show that the Cc/Glt NM spectrum includes most of the characteristic peaks of both curcumin and gelatin, namely, the peak for highly mixed vibrations of v (C=O), δ (CCC) and δ (CC=O), and aromatic v (CC) and v (CCH) of curcumin at 1511 cm^−1^ and the peak at 3302 cm^−1^ for the N–H stretching vibration of gelatin. However, some curcumin peaks were not detected in the Cc/Glt NM spectrum (e.g., the peak at 3507 cm^−1^ in the Cc/Glt NM spectrum), which suggests an intermolecular hydrogen bond between curcumin and gelatin. The amorphous separation of curcumin and noncovalent interaction between curcumin and gelatin prompts the sustained release of curcumin from Cc/Glt NMs, which was further validated by curcumin release experiments in vitro. As shown in Fig. 1f, Cc/Glt NM is capable of persistently maintaining curcumin concentration in the surrounding solution to as high as ~ 2 mg/ml for more than 120 h (please also see Additional file 1: Fig. S1).

### Somatic toxicity of curcumin/gelatin NMs

**Fig. 2:**
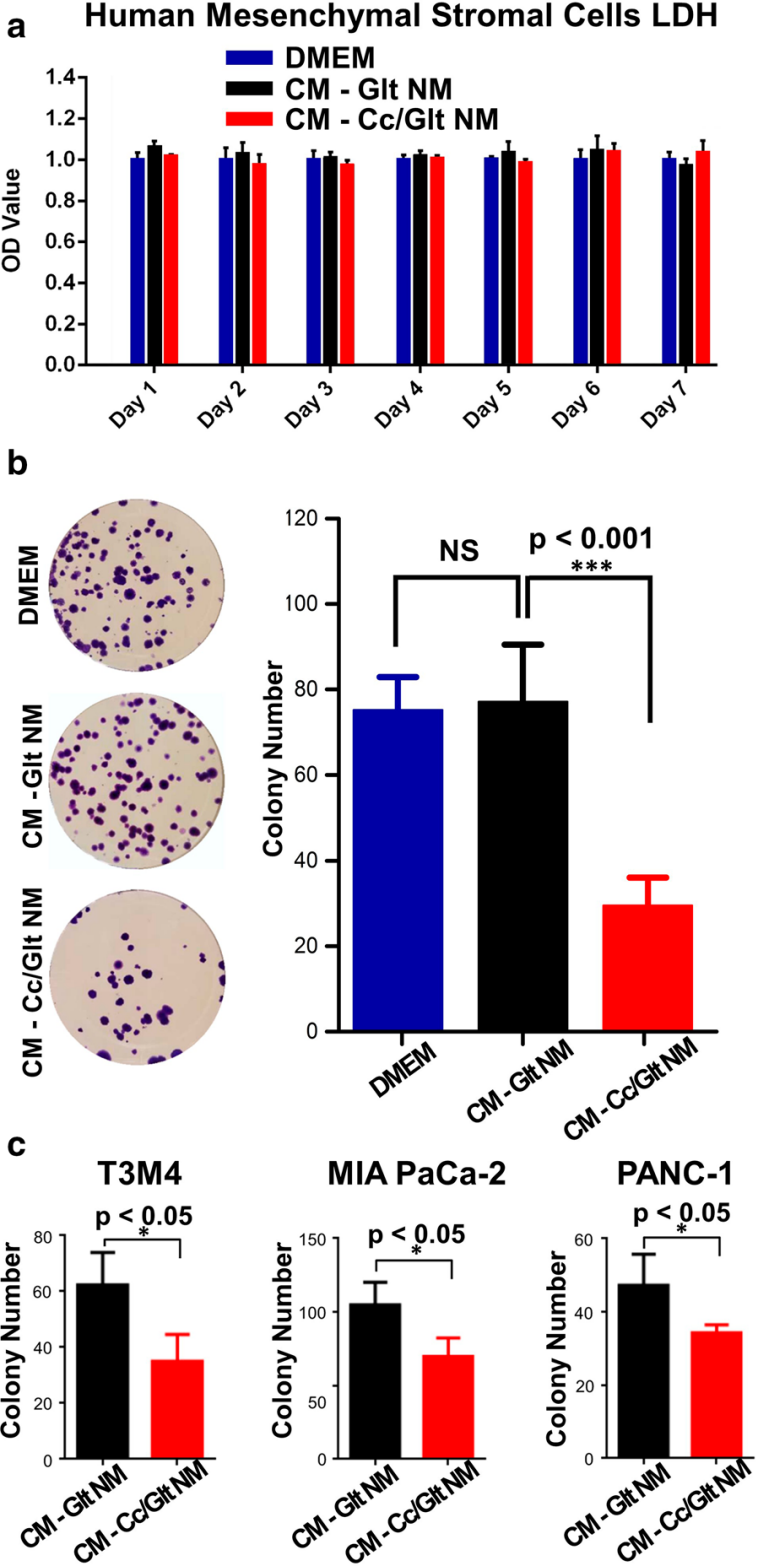
Cytotoxicity of curcumin/gelatin NMs on cultured cells. **a** LDH level was monitored for 7 days to asses cytotoxic effect of curcumin/gelatin NM on human mesenchymal stromal cells. As a result, the released curcumin exhibited no cytotoxicity to treated hMSCs; **b** The curcumin released from NMs inhibits murine PDAC cells’ proliferation as manifested by markedly decreased clone formation, which is verified by using three different types of human PDAC cells (**c**). (error bar: standard deviation)

According to the experimental results above, we can safely conclude that the method proposed here to prepare curcumin/gelatin-blended nanofibrous mats by electrospinning can dramatically enhance curcumin bioavailability. Although it has been widely established that curcumin is safe to normal cells and tissues, the very low amount of active curcumin available to act on those cells and tissues due to its extremely low bioavailability should not be neglected. Therefore, it is well necessary to further verify the harmlessness of released curcumin (when its solubility is markedly increased) on those cells and tissues. Hence, we performed lactate dehydrogenase (LDH) activity assay to evaluate the toxicity of Cc/Glt NMs on human mesenchymal stromal cells (hMSCs). LDH released from hMSCs cultured with CM-Cc/Glt NMs, CM-Glt NMs or control medium (DMEM) was measured. As shown in Fig. 2a, there was no significant difference in LDH level between cells treated with CM-Glt NMs, CM-Cc/Glt NMs or simply left untreated (control), indicating that Cc/Glt NM has no detectable toxicity to cells from normal tissue, even when the co-culture period lasts as long as seven days. The effects of Cc/Glt NMs or Glt NMs on the viability of hMSCs were also evaluated by MTT (3-(4, 5-dimethylthiazolyl-2)-2, 5-diphenyltetrazolium bromide) assay. The results showed that there was no significant change of cell proliferation at least in the first 6 days (Additional file 1: Fig. S2).

### Inhibited proliferation and induced apoptosis of pancreatic cancer cell in vitro

In order to validate the effect of curcumin in the Cc/Glt NM on PDAC cells, a well-established murine PDAC cell line 399 (with genotype p48^Cre/+^, Kras^G12D/+^, Tsc1^fl/+^) was used in the colony formation assay. After incubation with nano-curcumin for 72 h, murine PDAC cells’ proliferation was inhibited (Fig. 2b). The colony number decreased by approximately 60% in the group treated with conditioned medium of Cc/Glt NM (CM-Cc/Glt NM) compared to the group treated with conditioned medium of Glt NM (CM-Glt NM) and the control group (colony number in the group treated by DMEM vs. CM-Glt NM vs. CM-Cc/Glt NM: 75.2 ± 7.7 vs. 77.2 ± 13.3 vs. 29.6 ± 6.5). Moreover, the human PDAC cell lines T3M4, MIA PaCa-2 and PANC-1 showed similar results (Fig. 2c).

**Fig. 3:**
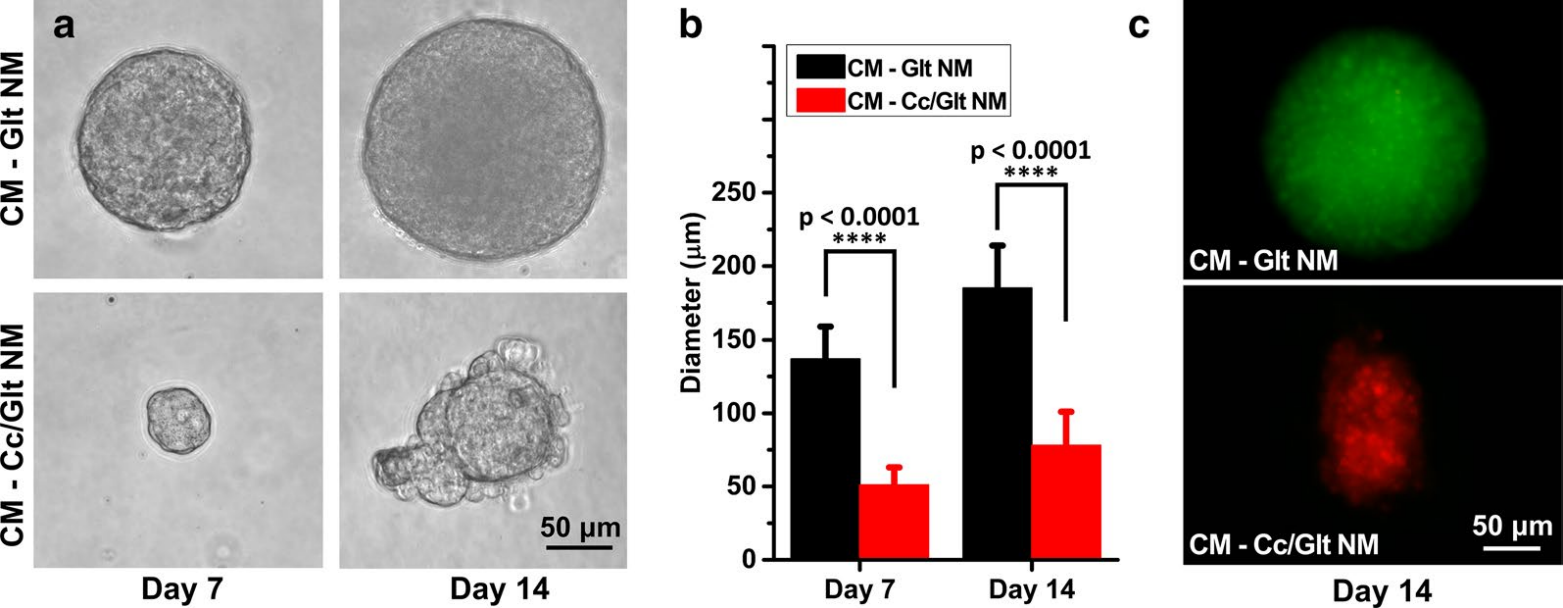
Proliferation inhibition effects of curcumin/gelatin-blended nanofibrous mats on pancreatic cancer cell clusters. **a** Phase contrast microscopy imaging of PDAC cell clusters on days 7 and 14 after coculture w/o curcumin; **b** the average sizes of the PDAC cell clusters on days 7 and 14 (error bar: standard deviation); and **c** fluorescence imaging of LIVE/DEAD-stained PDAC cell clusters on day 14

To further verify the effect of nano-curcumin against pancreatic cancer, 3D cultured cell clusters of PANC-1 were adopted as the in vitro tumor model for test. The size of the 3D cultured cell clusters co-cultured with CM-Cc/Glt NM or CM-Glt NM was tracked by phase contrast microscopy (Fig. 3a). The diameters of 10 cell clusters randomly selected from each group were measured on day 7 and 14 after nano-curcumin was added to the culture medium. The diameter of cell clusters co-cultured with CM-Glt NM dramatically increased to 137 ± 22 μm (in diameter) on day 7 and 185 ± 29 μm on day 14, while those subjected to CM-Cc/Glt NM treatment remained relatively small in size, exhibiting diameter of 51 ± 12 μm on day 7 and 78 ± 23 μm on day 14 (Fig. 3b), which proved the effectiveness of Cc/Glt NM in curbing pancreatic cancer cells’ proliferation. To further confirm the tumor suppressive activity of Cc/Glt NM, LIVE/DEAD fluorescent staining was used to investigate the survival state of Cc/Glt NM or Glt NM-treated cell clusters on day 14. As shown by Fig. 3C, most cells in the CM-Glt NM co-cultured cluster emit green fluorescence (alive); in contrast, cell clusters in Cc/Glt NM treated group gives off nearly entire red fluorescence (dead), denoting cell death or damage of almost all cancer cells in the cluster. This finding prompted us to conclude that Cc/Glt NM may actively induce pancreatic cancer cell apoptosis.

### Bip activation and p-STAT3 inhibition

**Fig. 4:**
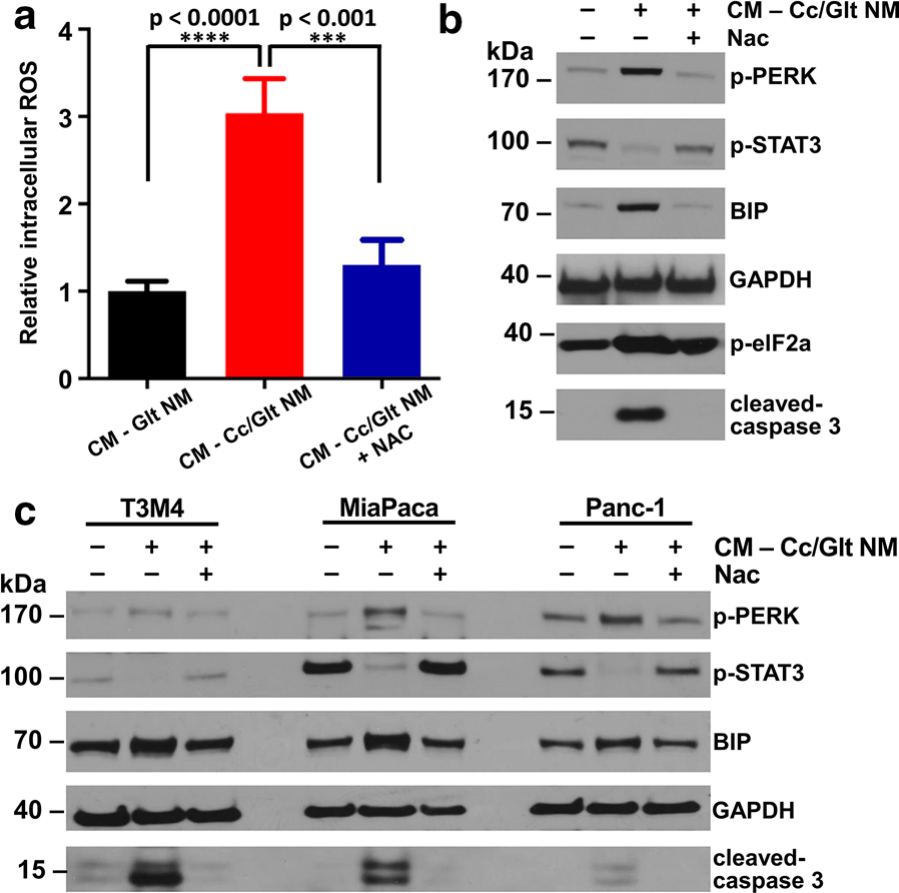
Molecular mechanism study of nano- curcumin against pancreatic cancer. **a** Intracellular ROS levels of murine PDAC cells changed by the curcumin released from Cc/Glt NMs (error bar: standard deviation); **b** western blot analysis of the expression level changes of cleaved-caspase 3, phospho-PERK, BIP, phospho-elF2α and phospho-STAT3 in murine PDAC cells; and **c** western blot analysis of the expression level changes of cleaved-caspase 3, phospho-PERK, BIP and phospho-STAT3 in three types of human PDAC cells

To further verify the effects of Cc/Glt NMs on the induction of PDAC cell apoptosis and explore the possible underlying mechanisms, we first measured the intracellular ROS level after Cc/Glt NM treatment. As Fig. 4a shows, reactive oxygen species (ROS) production in Cc/Glt NM treated group was significantly higher (threefold) than that in Glt NM treated group. Besides, western blotting results showed that after being treated with Cc/Glt NM, the expression of cleaved-caspase 3, phospho-PERK, BIP, and phospho-elF2a in murine PDAC cells was upregulated, while the expression of phospho-STAT3 was decreased. Meanwhile, exogenous supplemental *N*-acetyl-l-cysteine (NAC), a specific ROS scavenger, can rescue the alteration in cells treated with Cc/Glt NM (Fig. 4a, b). Therefore, it is safe to conclude that Cc/Glt NM can induce pancreatic cancer cell apoptosis by increasing ROS production to trigger the downstream signaling of ER stress, namely, Bip/p-PERK/p-elF2a pathway and inhibit the phosphorylation of STAT3. The same results were observed in three other types of human PDAC cells, T3M4, MIA PaCa-2 and PANC-1 (Fig. 4c). Thus, we proved that Cc/Glt NM could promote apoptosis by stimulating ER stress and regulating STAT3 phosphorylation in pancreatic cancer cells. These results may not only help clarify the underlying molecular mechanisms of nano-curcumin against pancreatic cancer but also provide reasonable evidence for its translation as coping strategy to deal with this fatal malignancy in the clinic.

### In vivo pancreatic cancer therapy

**Fig. 5:**
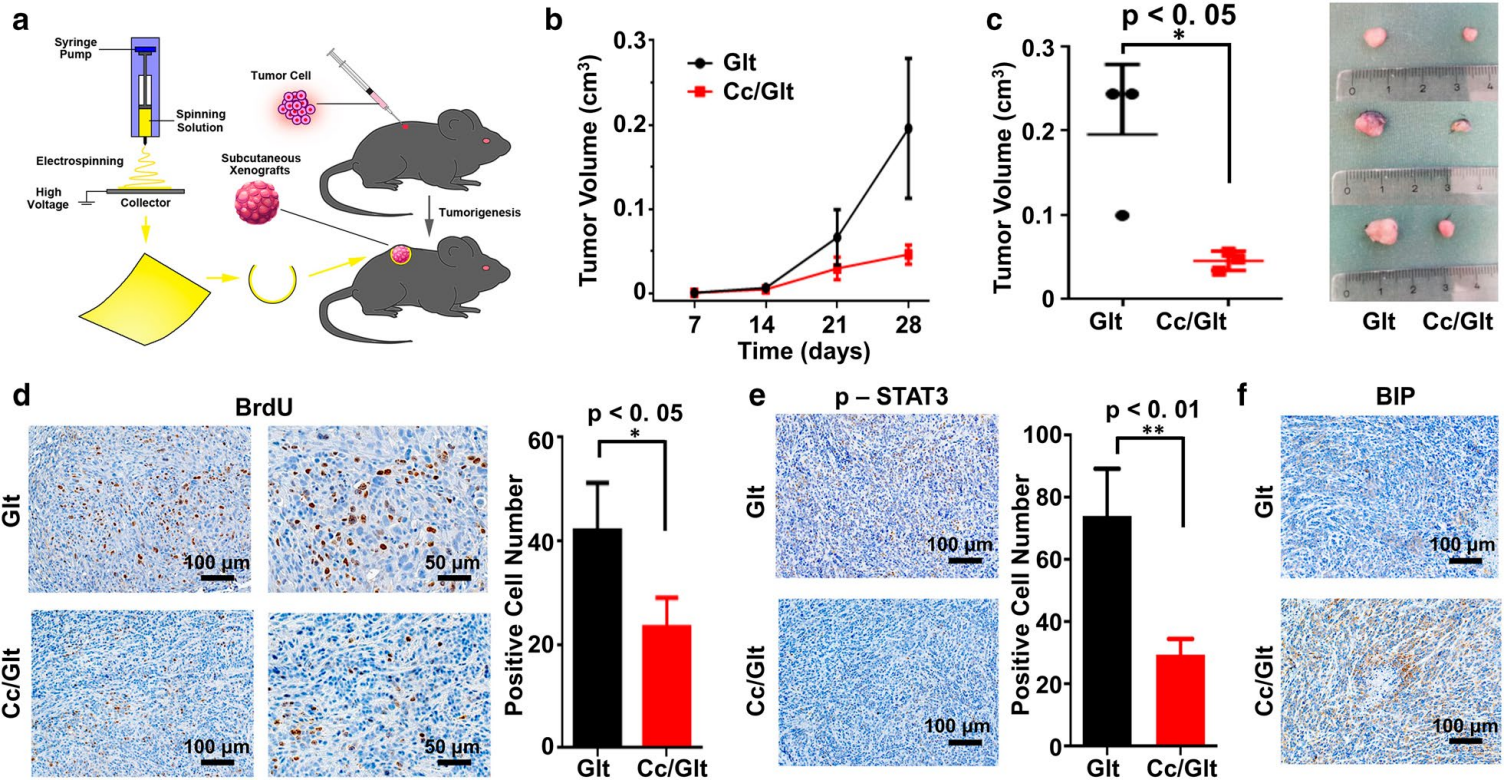
Antitumor effects of curcumin/gelatin-blended nanofibrous mats in a mouse model of PDAC. **a** The flow diagram of the antitumor effect study of Cc/Glt NMs; **b** the tumor volume change over time with the Cc/Glt NMs or Glt NMs applied on day 14; **c** the photographs and statistical volume results of the PDAC tumors after Cc/Glt NMs or Glt NMs were applied for 14 days; **d** BrdU labeling of the tumor sections to detect the proliferation of active cells after 14 days of treatment with Cc/Glt NMs; **e** the phospho-STAT3 expression levels in the tumor after 14 days of treatment with Cc/Glt NMs; and **f** the BIP expression levels in the tumor after 14 days of treatment with Cc/Glt NMs. (error bar: standard deviation)

In order to determine the feasibility of curcumin/gelatin-blended nanofibrous mat against pancreatic cancer in vivo, 1 × 10^6^ murine PDAC cells were inoculated into parallel bilateral flank of C57Bl6 mouse subcutaneously. After two weeks, as visible mass formed subcutaneously, Cc/Glt NM or Glt NM was embedded around the surface of the xenograft closely (Fig. 5a). After the topical application of Cc/Glt NM, tumor growth was observed to remarkably decreased (Fig. 5b), as tumor volume was significantly smaller than that in the control group (Fig. 5c). Moreover, there were significantly fewer BrdU-positive cells detected in the tumorous tissues treated by Cc/Glt NM (Fig. 5d), which indicated a marked inhibition of cancer cell proliferation after Cc/Glt NM embedding. Meanwhile, immunohistochemistry analysis further confirmed that after being exposed to Cc/Glt NM, the expression of Bip in the tumor was upregulated, verifying that ER stress was activated (Fig. 5f), which further reduced phosphorylation of STAT3 in tumors (Fig. 5e). Taken together, these results suggest that Cc/Glt NM may act as a promising topical agent against PDAC in the clinic, as through local delivery, nano-curcumin exhibits favorable pharmacological properties to induce and cytocidal effect on both PDAC cell and xenograft tumor, respectively.

## Discussion

Many traditional medicines (such as traditional Chinese medicine and traditional Indian medicine) have been verified for their safety after a long period of practice [57, 58]. Additionally, recent studies have purified the active ingredients and revealed the biological functional mechanisms [59, 60]. One successful example is curcumin, which is the natural polyphenol and principal curcuminoid found in the rhizome of a well-documented medicinal plant (turmeric). Experimental studies have shown that curcumin has many beneficial pharmacological effects such as anti-cancer, anti-inflammatory, anti-mutagenic and anti-coagulant, to name but a few, which makes it a research hotpot over decades [39, 40, 61–63]. Therefore, it is considered to be effective treatment for cancer, rheumatoid arthritis, wound healing, cardiovascular diseases and so on [64, 65]. However, like many other naturally derived active compounds from traditional medicinal plant, the actual performance of curcumin in clinical practice are doubted [46, 48, 66]. This is mainly due to its poor solubility and low bioavailability [42]. Although curcumin analogs have been developed to improve its biocompatibility and bioavailability, among which the molecular mechanisms interpreting pancreatic cancer cell apoptosis by some of these soluble curcumin analogs have been outlined, convincing experimental evidence are still required pertaining to their biosafety and mechanism of action compared to natural curcumin before they can be further translated in the clinic. Recent advances in material science indicate that the development of nano-sized delivery systems is helpful to overcome curcumin’s own defects, which has shed some new lights for its clinical translation [42]. As a tentative attempt, we made curcumin/gelatin-blended nanofibrous mat capable of controlled release of incorporated curcumin by electrospinning (Fig. 1a), which largely improved curcumin’s bioavailability as it has been physically changed its crystal state into nanoscale solid dispersion (Fig. 1d).

Material characterization indicate that curcumin encapsulated in NMs obtained by this method has an unchanged molecular structure as curcumin powder purified from native turmeric (Fig. 1e). Most importantly, by transforming native curcumin to the prepared Cc/Glt NMs, curcumin can be continuously released into the surrounding solution for days (Fig. 1f), which preserves its stability to the greatest extent. Also, the released curcumin had no obvious toxicity to somatically derived normal cells but has remarkable cytocidal effects on pancreatic cancer cells of different origin (human and murine) (Figs. 2, 3). Therefore, we could further scrutinize how nano-curcumin acts against PDAC and found that it raises ROS level inside pancreatic cancer cells to activate ER stress signaling and finally inhibit the phosphorylation of downstream effector STAT3, leading to cell apoptosis in the end (Fig. 4). This was verified by an in vivo mouse model of Xenograft tumors that two weeks after the application of Cc/Glt NM, the growth of the tumor was significantly inhibited, which is consistent with markedly decreased cell proliferation in these tumor tissues. Therefore, we attribute these observations to changes in ER stress signaling and phosphorylation levels of STAT3 induced by nano-curcumin (Fig. 5).

Prior reports have shown that curcumin produces inhibitory effects on multiple targets, such as inflammatory cytokines, growth factors, anti-apoptotic proteins, and transcriptional factors, which further suppress inflammation, angiogenesis, proliferation and apoptosis [67]. Dhillon and the co-workers reported clinical response of curcumin to repress phosphorylation of constitutive STAT3 by daily oral administration in pancreatic cancer patients [46]. Therefore, our findings that nano-curcumin inhibits p-STAT3 is in consistence of previous evidence. Besides, it is interesting to note that we also found that nano-curcumin activated Bip/PERK/elf2a axis to induce apoptosis and impair proliferation, which further illustrated its multiple molecular targets of anti-tumor role. However, more works are still needed to further elucidate its potential mechanisms.

In view of above, we have provided a feasible approach to effectively improve the bioavailability of curcumin. To be more precisely, the curcumin encapsulated inside the NMs with enhanced solubility and stability by the current method demonstrated significant impact on PDAC cells both in vitro and in vivo. More importantly, the effectiveness of the Cc/Glt NM to successfully suppress tumor growth has also been proven by animal experiments, which provides meaningful evidence for its potential application in later clinical settings. It is noteworthy that although our method has improved the bioavailability of curcumin through sustained release, a more precise drug transportation and targeted delivery of curcumin by taking advantage of gelatin’s good biodegradability and non-antigenicity is yet to be explored. Hopefully, this can be solved by preparing smaller Cc/Glt NM for direct injection with more accuracy or by chemical modification to allow Cc/Glt NMs to bind to targeted tissues or cells. Meanwhile, the macroscopic Cc/Glt NMs may be applicable as a potential chemo-preventive agent or a novel adjuvant treatment to eliminate peripheral residual cancer cells after ablative surgery for pancreatic cancer.

At the same time, the most readily available approach to home in tumor for targeted therapy is to deliver the drug (or its active ingredients) directly to targeted area and allow it to stay there at a proper concentration for proper time. In this work, the preparation of drug loaded nanofibrous mat directly disperses the drug into a solid state membrane so that when the membrane is topically applied to the tumor region, targeted drug delivery was achieved in a sense. On the other hand, it has been found that the dissolution rate of drug particle will markedly increase when its surface area to volume ratio is increased. Therefore, the blended nanofibrous mat here (by mixing curcumin with gelatin) has greatly increased curcumin dissolution by increasing its surface area to volume ratio. Meanwhile, FTIR analysis also prompted that curcumin was incorporated into the fiber only with non-covalently bounding, so it can mildly return to the environmental solution by diffusion in a sustainable manner. Since the enhanced solubility and the ability of sustained release are both driven by physical factors without the need of any specific covalent bond or other interactions between the drug and the carrier, our method to prepare drug loaded nanofibrous mat may provide a general solution to improve the bioavailability of other natural compounds and traditional medicines, in other words, it is reasonable to assume that this strategy might be well suitable to be adopted for the exploitation of other medical compounds with low bioavailability—but more investigations are yet to be conducted.

## Conclusions

In this study, curcumin loaded gelatin nanofibrous mats were prepared by electrospinning. Curcumin encapsulated inside the NMs could therefore be sustainedly released to reach a certain level in the environmental solution and last for days. The controlled release significantly improved the bioavailability of curcumin, which was proven by in vitro and in vivo assays. Notably, upon topical application of the curcumin/gelatin-blended nanofibrous mat, the growth of the xenografts tumor in the animal model were seen evidently suppressed. Therefore, the underlying mechanisms were further scrutinized. It was found that the released curcumin can activate the production of intracellular ROS, which induced ER stress and decreased the phosphorylation of STAT3. In conclusion, the dispersion of curcumin into the NMs through electrospinning might be a promising approach for combination therapy against pancreatic cancer and many other neoplastic diseases by virtue of its safety, potency and cost-effectiveness.

## Methods

### Electrospinning and cross-linking

One gram of gelatin (Sigma-Aldrich, Saint Louis, China) and 0.1 g of curcumin (Sigma-Aldrich, Saint Louis, China) were dissolved in 10 ml of trifluoroethanol (Sigma-Aldrich, Saint Louis, China) and stirred at room temperature for 2 h until they were completely dissolved to obtain a uniform spinning solution. For electrospinning, the spinning solution was loaded into a 10 ml syringe with a 20-gauge nozzle. The syringe was placed 10 cm from the collector (aluminum foil wrapped copper plate) and the feed rate was set to 1.5 ml per hour controlled by a syringe pump (Cole-Parmer, US). The DC voltage was set at 15 kV. The residual organic solvent of the prepared sample was removed by vacuum drying, and then the sample was processed by a cross-linking process. Namely, a 25% glutaraldehyde solution (Sigma-Aldrich, Saint Louis, China) and ethanol mixture (1% V/V) were placed together with the prepared nanofibrous mat in a vacuum desiccator at 24 °C for 24 h. After that, the cross-linked NM was rinsed with ultrapure water and lyophilized for 24 h. A gelatin NM without curcumin (Glt NM) was made in the same way as control for the subsequent experiments.

### Nanofiber characterization

A gold film was first sputter coated on the prepared NM. Then, the coated samples were imaged by a scanning electron microscope (JSM-5600LV, JEOL, Japan) with an acceleration voltage of 8–10 kV.

The NM samples were subjected to X-ray diffraction (XRD) measurements using an X-ray diffractometer (D8 ADVANCE, Bruker, Germany). CuKα radiation was in the 2θ range of 0–60°, and the operating voltage and current were 40 kV and 300 mA, respectively. The scan rate was 1° (2θ)/min.

Prepared NM samples were subjected to Fourier transform infrared spectroscopy (FTIR) using a Fourier transform infrared spectrometer (Nicolet 6700, Thermo Fisher Scientific, US) with a scanning resolution of 2 cm^−1^ and a scanning range of 500–4000 cm^−1^.

A piece of NM was placed under a fluorescence microscope (Ti-E, Nikon, Japan) with a 40× objective lens. According to the fluorescence spectrum characteristics of curcumin, FITC filter blocks (Ex: 480/30 nm, Em: 535/45) were selected for imaging.

### In vitro curcumin release

A 1.5 cm × 1.5 cm Cc/Glt NM or 0.1 g of curcumin powder (corresponding to the same curcumin content as the 1.5 cm × 1.5 cm Cc/Glt NM) was immersed into 50 ml of phosphate-buffered saline (PBS, pH 7·4, 37 °C). Two hundred microliters of solution was removed at defined intervals for curcumin concentration, and an equal volume of PBS was added back to the original system to maintain a total volume of 50 ml. A high-performance liquid chromatography (HPLC, 1200, Agilent, US) system combined with a quadrupole mass spectrometer (API-4000, AB SCIEX, US) system was employed for the sample analysis. The HPLC system was operated with a column (4.6 × 150 mm, 3.5 μm, Eclipse XDB-C18, Agilent, US) and a 300 μl/min mobile phase solution of water and methanol. The declustering potential, collision energy and collision cell exit potential were set to − 70, − 50 and − 12 V, respectively. Three lots of identical samples were evaluated at each scheduled time period, and the average number time lapse curve was plotted.

### Conditioned medium preparation

A 2 mm × 2 mm Cc/Glt NM or Glt NM was immersed into the 1 ml DMEM culture medium without FBS and incubated at 37 °C for 72 h. The conditioned medium of Cc/Glt NM or Glt NM was prepared by mixing the leaching solution with an appropriate amount of DMEM cultured medium with FBS.

### Cell cytotoxicity and viability assays

Human mesenchymal stromal cells (hMSCs) (SCSP-405, cells were kindly provided by the Stem Cell Bank, Chinese Academy of Sciences) were seeded into a 96-well plate at a density of 5 × 10^3^ cells per well in 100 μl of mesenchymal stem cell basal medium with a bone marrow-mesenchymal stem cell growth kit (PCS-500-030, ATCC, US). Twenty-four hours later, the culture medium was replaced with CM-Glt NM or CM-Cc/Glt NM. The lactate dehydrogenase (LDH) activity assay and MTT assay was performed every day, according to the manufacturer’s instructions (Beyotime Biotechnology, Shanghai, China), and data were collected with a microplate reader (Cytation 5, BioTek, US).

### PDAC cell culture

The murine PDAC cell line 399 (generated from the tumor developed in a genetically engineered mouse genotyping p48^Cre/+^; LSL-Kras^G12D/+^; Tsc1^fl/+^ as published [56], a kind gift from Dr. Bo Kong, Department of Surgery, Klinikum Rechts der Isar, Technical University of Munich) was cultured in DMEM high glucose medium (Gibco, Waltham, US) supplemented with 10% FBS (Gibco, Waltham, US), 100 U/ml penicillin and 100 µg/ml streptomycin (Gibco, Waltham, US) at 37 °C and 5% CO_2_. The human PDAC cell lines PANC-1, T3M4 and MIA PaCa-2 were provided by the Stem Cell Bank, Chinese Academy of Sciences and cultured under the same conditions as the above murine PDAC cells.

### Colony formation

In order to evaluate the effect of the curcumin released from Cc/Glt NMs on PDAC cells, PDAC cells (3 × 10^2^) were seeded in 6-well plates and continuously cultured in CM with or without curcumin. After 7 days, the cells were fixed with methanol and stained with 10% crystal violet (Beyotime Biotechnology, Shanghai, China). The colony number (containing more than 50 cells) was counted by three independent researchers under a microscope.

### In vitro pancreatic cancer cell cluster killing

The human PDAC cell lines T3M4, MIA PaCa-2 and PANC-1 and murine PDAC cells were cultured as described above. A total of 2 × 10^5^ cells were seeded with 10 ml of precooled Matrigel solution (Sigma-Aldrich, Saint Louis, China) and gently pipetted in an ice bath until the cells were fully mixed with the Matrigel solution. A drop of 1.5 ml of Matrigel solution containing ~ 3 × 10^4^ cells was then added to a 6-well cell culture plate. After the Matrigel was solidified at 37 °C for 1 h, 3 ml of DMEM was added to each well and cultured at 37 °C for 24 h, and then the culture medium was replaced with CM-Glt NM or CM-Cc/Glt NM. The cell morphology was observed every day. After 14 days, cell clusters were stained with a LIVE/DEAD viability kit (Thermo Fisher Scientific, Waltham, US) and were imaged by fluorescence microscopy to visualize the dead and live cell. Three independent samples were chosen for analysis from both the Cc/Glt NM and Glt NM groups, and 10 cell clusters in each sample were randomly selected and measured.

### Intracellular ROS measurement

PDAC cells (1 × 10^4^) were seeded in a black 96-well plate and cultured with conditioned medium of nano-curcumin for 24 h. Then, 1 mM DCFH-DA was added to the cells followed by incubation at 37 °C for 1 h. Then, the level of intracellular ROS was determined by using an OxiSelect Intracellular ROS assay kit (Cell Biolabs, San Diego, US).

### Western blot analysis

The human PDAC cell lines T3M4, MIA PaCa-2 and PANC-1 and murine PDAC cells were co-cultured with CM-Cc/Glt NM or CM-Glt NM. After 2 days, the total protein was prepared with diluted cell lysis buffer (Beyotime Biotechnology, Shanghai, China) supplemented with protease inhibitor (Beyotime Biotechnology, Shanghai, China) and phosphatase inhibitor (Beyotime Biotechnology, Shanghai, China). The expression of BiP, p-PERK, p-STAT3, p-elF2a, and cleaved-caspase 3 was examined by using antibodies from Cell Signaling Technology (Frankfurt am Main, Germany), i.e., anti-BiP antibody (3177), anti-phospho-PERK (Thr980) antibody (3179), anti-phospho-eIF2a (Ser51) antibody (9721) and anti-phospho-STAT3 antibody (9145). GAPDH (sc-25778, Santa Cruz Biotechnology, Santa Cruz, US) was used as a housekeeping gene.

### Tumor xenograft model

A total of 1 × 10^6^ tumor cells were subcutaneously transplanted into both sides of the backs of 8-week-old wild-type mice. On day 14, surgery was performed to place the Cc/Glt NM or Glt NM tightly between the subcutaneous xenograft and muscle. Tumor volumes were measured and calculated every week. On day 28, the mice were sacrificed, and the xenografts from both sides were collected. The long and short diameter of each xenograft was measured to calculate tumor volume by its average diameter. The results were stated as mean ± standard error of the mean, and two-sample t-test was used, statistical significance was set at p < 0.05. The study was performed under a protocol approved by the Institutional Animal Care and Use Committee of Shanghai Jiao Tong University (Document number: 2020038).

### Immunohistochemistry and positive cell calculation

Tissue sections were deparaffinized and rehydrated, and antigen retrieval was performed with either citrate buffer or proteinase K (Beyotime Biotechnology, Shanghai, China). After endogenous peroxidase and nonspecific binding blocking, sections were incubated with anti-cleaved-caspase 3, anti-BiP, and anti-BrdU antibodies (ab6326, Abcam, Cambridge, US). After incubation with rabbit HRP-labeled anti-rat antibody or goat HRP-labeled polymer anti-rabbit antibody (ab6734, ab214880, Abcam, Cambridge, US), a color reaction was performed with a liquid DAB+ substrate chromogen system. Then, the sections were counterstained with Mayer’s hematoxylin, dehydrated and mounted. For calculation of the positively stained cells, five fields were randomly selected and images were taken with a microscope (Ti-E, Nikon, Japan). The number of positively stained cells was counted by two independent researchers using ImageJ software (NIH, US).

### Statistical analysis

The data was analyzed by using OriginPro 9.1 software (OriginLab, Northampton, US). Each data shown here was from at least three independent experiments and expressed as mean ± standard deviation (SD). Two-sample t-test was used to conduct the comparison of the two groups’ means. p-values of higher than 0.05 were considered not statistically significant.

## Supplementary information

**Additional file 1: Fig. S1.**. Fluorescence imaging of the curcumin/gelatin-blended nanofiber mats after being immersed in PBS over time. ** Fig. S2.** MTT cell viability assay for human mesenchymal stromal cells treated with CM-Cc/Glt NM or CM-Glt NM (error bar: standard deviation).

## Data Availability

The datasets used and/or analyzed during the current study are available from the corresponding author on reasonable request.

## Abbreviations

PDAC: Pancreatic adenocarcinoma
ROS: Reactive oxygen species
ER stress: Endoplasmic reticulum stress
STAT3: Signal transducer and activator of transcription 3
NMs: Nanofibrous mats
Cc/Glt NMs: Curcumin/gelatin NMs
Glt NMs: Gelatin NMs
XRD: X-ray diffraction
FTIR: Fourier transform infrared spectroscopy
hMSCs: Human mesenchymal stromal cells
LDH: Lactate dehydrogenase
MTT: 3-(4, 5-Dimethylthiazolyl-2)-2, 5-diphenyltetrazolium bromide
CM: Conditioned medium
CM-Cc/Glt NM: Conditioned medium of Cc/Glt NM
CM-Glt NM: Conditioned medium of Glt NM
NAC: *N*-Acetyl-l-cysteine
OD: Optical density
DMEM: Dulbecco's modified Eagle medium
FBS: Fetal bovine serum
