# Supplementary material for: Topically applicated curcumin/gelatin-blended nanofibrous mat inhibits pancreatic adenocarcinoma by increasing ROS production and endoplasmic reticulum stress mediated apoptosis
Source: J Nanobiotechnology. 2020 Sep 5;18:126. doi: 10.1186/s12951-020-00687-2 (PMC7487882; doi:10.1186/s12951-020-00687-2)
Supplement: Supplementary file 1 — Additional file 1: Fig. S1.. Fluorescence imaging of the curcumin/gelatin-blended nanofiber mats after being immersed in PBS over time. Fig. S2. MTT cell viability assay for human mesenchymal stromal cells treated with CM-Cc/Glt NM or CM-Glt NM (error bar: standard deviation). [file 12951_2020_687_MOESM1_ESM.pdf]

Additional file 1

Figure S1.jpg

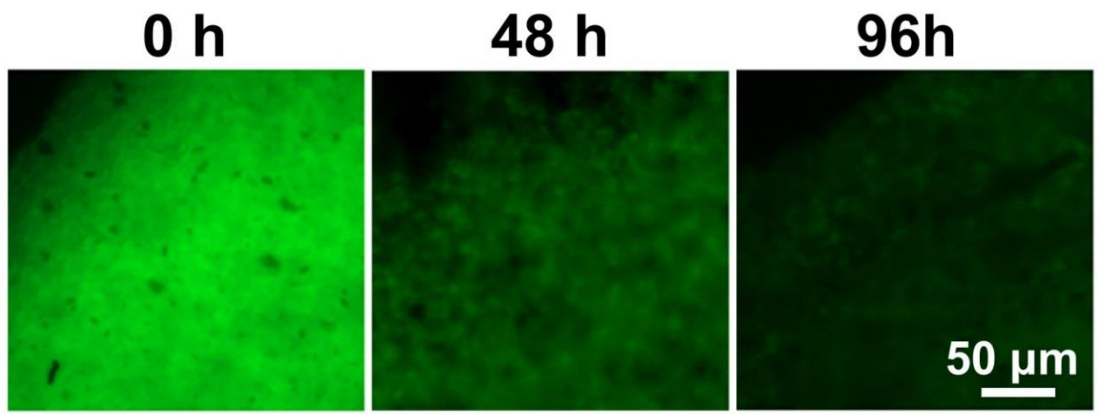

Figure S1. Fluorescence imaging of the curcumin/gelatin-blended nanofiber mats after being immersed in PBS over time.

Figure S2.jpg

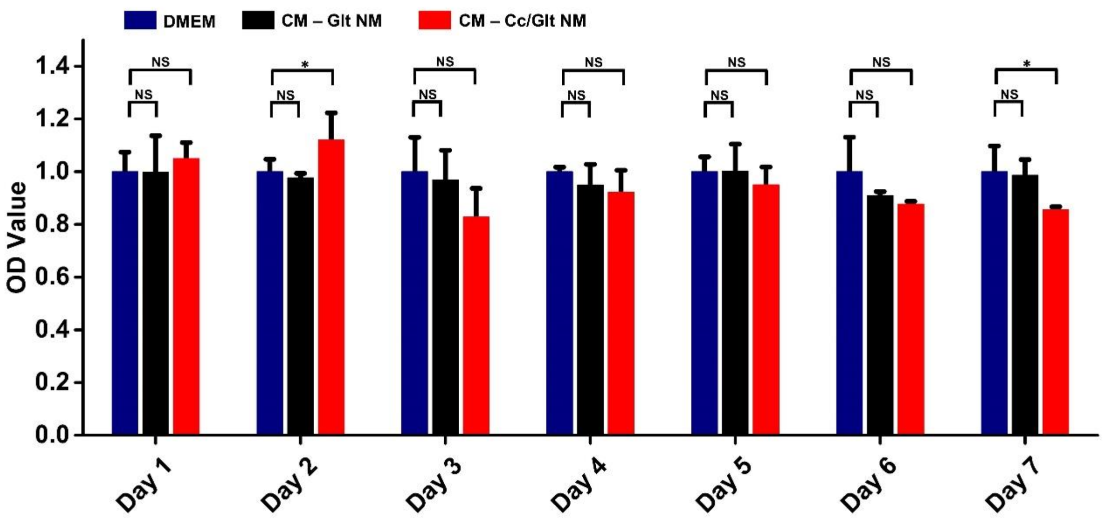

Figure S2. MTT cell viability assay for human mesenchymal stromal cells treated with CM – Cc/Glt NM or CM – Glt NM (error bar: standard deviation).
